# Artificial Oxidation: A Major Challenge in Implementing Multi-Attribute Methods for Therapeutic Protein Analysis

**DOI:** 10.3390/ph19040528

**Published:** 2026-03-25

**Authors:** Yaokai Duan, Michael Lanzillotti, Dylan L. Riggs, Albana Nito, Junnichi Mijares, Amanda Helms, Carl Ly, Kevin Millea, Xingwen Li, Hao Zhang, Zhongqi Zhang

**Affiliations:** 1 Process Development, Amgen Inc., Thousand Oaks, CA 91320, USA; yduan@amgen.com (Y.D.); jmijar01@amgen.com (J.M.); 2 Process Development, Amgen Inc., Cambridge, MA 02142, USA; mlanzill@amgen.com (M.L.); driggs01@amgen.com (D.L.R.); ahelms01@amgen.com (A.H.); cly02@amgen.com (C.L.); kmillea@amgen.com (K.M.); xingwenl@amgen.com (X.L.); zhanghao.wustl@gmail.com (H.Z.); 3 Quality, Amgen Inc., Thousand Oaks, CA 91320, USA; anito@amgen.com

**Keywords:** multi-attribute method, therapeutic proteins, peptide mapping, methionine oxidation, artificial oxidation, LC-MS, metal-induced oxidation

## Abstract

**Background/Objectives**: Mass spectrometry-based multi-attribute methods (MAM) have the potential to transform therapeutic protein analysis by enabling comprehensive monitoring of multiple quality attributes in a single assay. However, the widespread adoption of MAM is hindered by significant challenges, most notably artificial oxidation during sample preparation and analysis. This report summarizes long-term operational observations and several case studies that substantiate this concern. **Methods**: A tryptic digest, high-resolution LC-MS MAM workflow was applied to an Fc-fusion protein and multiple antibody-based therapeutics, with a frozen reference standard analyzed in each run for system suitability and longitudinal trending. Oxidation excursions were investigated by comparing laboratories, consumables, LC-MS configurations, and other method parameters. **Results**: In a seven-year trending record, apparent total methionine oxidation in the Fc-fusion protein reference standard showed an abrupt, sustained increase (up to ~5-fold); the shift was traced to a specific bag of microcentrifuge-tubes used during buffer exchange and resolved after those tubes were discontinued. In an antibody–drug conjugate, observed methionine oxidation was strongly influenced by the sample preparation procedure. In other antibodies, variability of observed methionine oxidation was attributed to on-column oxidation, which produced a broad and noisy peak that interferes with automated peak integration. EDTA flushing reduced this feature, implicating exposure to metal ions. **Conclusions**: While advances continue to address many MAM challenges, artificial oxidation remains unpredictable and constitutes a major obstacle to robust implementation in regulated QC environments. Enhanced control strategies and further research are urgently needed to ensure reliable therapeutic protein analysis. Such control strategies include consumable qualification and change control, system suitability/trending using a reference standard, metal management across LC flow path/column lifecycle, reduction of trifluoracetic acid (TFA) exposure, data analysis to safeguard excessive on-column oxidation, etc.

## 1. Introduction

The structural characterization of therapeutic proteins is a cornerstone in the development and quality assurance of biopharmaceuticals. Traditionally, chromatographic and electrophoretic techniques have been employed to monitor individual structural attributes by separating molecular species based on charge, hydrophobicity, or size. However, these methods are limited in that they often track one attribute per assay and lack specificity regarding the exact structural changes in each attribute, typically quantifying “peaks” rather than distinct molecular species.

The advent of mass spectrometry (MS)-based multi-attribute methods (MAM) has revolutionized this landscape. By leveraging liquid chromatography-mass spectrometry (LC-MS) following protease digestion, MAM enables the simultaneous monitoring of hundreds of molecular species, allowing for comprehensive attribute profiling in a single run [1,2,3,4,5,6,7,8,9,10]. This specificity is essential for distinguishing critical quality attributes from non-critical ones and has led to the widespread adoption of MAM and similar approaches for therapeutic protein characterization and quality monitoring [8,9].

Despite these advances, the deployment of MAM continues to present considerable challenges. These challenges must be properly addressed to enable widespread application of MAM, particularly in the quality-control arena. Aside from widely discussed logistical and regulatory challenges [9,10,11], technical challenges [7] include issues such as complex sample preparation, artificial modifications, instrument-to-instrument variability, and the intricacies of data analysis. However, we believe that the most formidable and persistent obstacle, although not widely discussed, is artificial oxidation occurring during sample preparation and analysis. Artificial oxidations are any oxidations introduced after sampling, such as during sample preparation, chromatographic separation, and electrospray ionization. Unlike other challenges, artificial oxidation remains largely unsolved and poses a significant hurdle to the reliable and timely release of clinical and commercial biotherapeutics. Compared to other artificial modifications such as deamidation, isomerization, succinimide formation/hydrolysis, etc., oxidation is particularly problematic because it is driven by trace metals, trace photosensitizers, light exposure, consumable leachables, and ion-source phenomena that are highly unpredictable and difficult to fully control.

In this article, we present long-term observations and representative case studies of oxidation artifacts across multiple therapeutic proteins during various stages of MAM analysis. We highlight why we believe artificial oxidation represents the most critical challenge to overcome for the successful implementation of MAM and emphasize the urgent need for solutions to this persistent artifact.

## 2. Results

### 2.1. Artificial Oxidation During Sample Preparation

We developed and implemented an MAM protocol to monitor several attributes of an Fc-fusion protein, including Met oxidations, Asn deamidations, clippings, and O-glycosylation, utilizing tryptic digestion and high-resolution LC-MS analysis [5]. The method has been used to monitor these attributes over a 7-year period during clinical development of the Fc-fusion protein. A frozen reference standard stored at −70 °C was analyzed in parallel with every run for system suitability and longitudinal trending.

**Figure 1:**
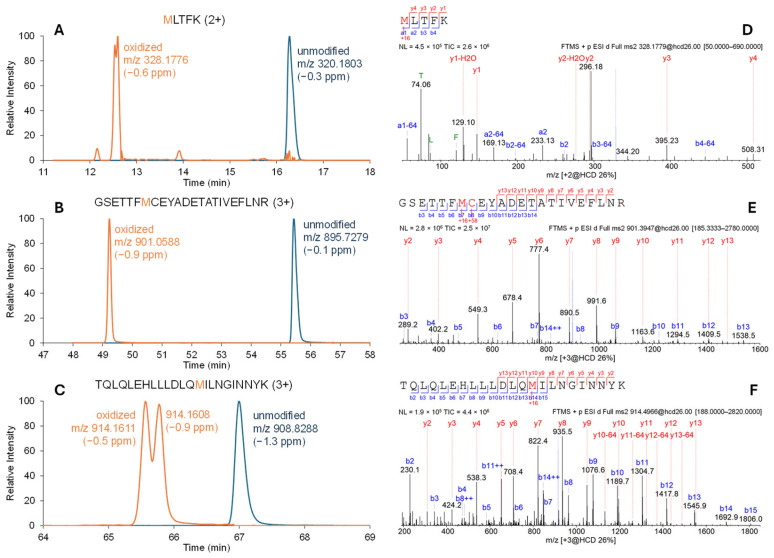
XICs (**A**–**C**) for three methionine-containing peptides and their oxidized forms from the tryptic digest of the Fc-fusion protein. Each XIC is normalized to its highest intensity. Theoretical monoisotopic *m*/*z* are 320.1804, 895.7280, and 908.8300, respectively for the three unmodified peptides, and 328.1778, 901.0596, and 914.1616, respectively for the three oxidized peptides. MS/MS evidence for the identification of the three oxidized peptides are shown in (**D**–**F**) for peptide (**A**), (**B**), and (**C**), respectively. Presence of neutral loss of 64 Da in the MS/MS indicates the presence of oxidized methionine. MS/MS of the two oxidized peaks in peptide (**C**) are essentially identical (only one is shown).

The Fc-fusion protein contains seven methionine residues, among which three were considered critical and their oxidations were monitored by the MAM method. The three methionine oxidation sites are distributed across three tryptic peptides. Methionine sulfoxides were monitored for all three peptides as methionine sulfone species were negligible. Figure 1 shows the extracted-ion chromatograms (XICs) of each oxidized peptide. Some of the oxidized peptides show two closely eluted peaks, as expected due to the presence of diastereomers after oxidation of the methionine side chain [12]. Total methionine oxidation (sum of the observed oxidation levels of the three methionine sites) was recorded for trending purposes.

**Figure 2:**
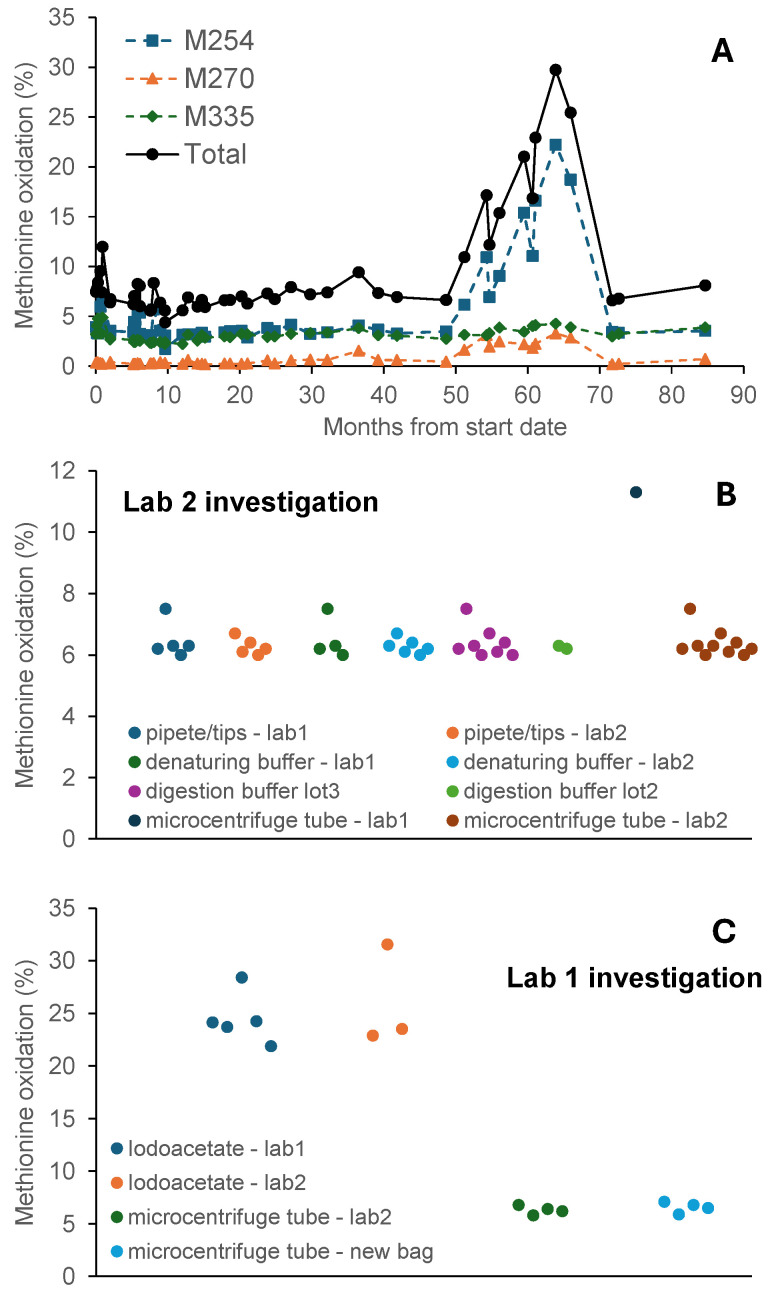
Seven-year trend (performed in Lab 1) showing an increase in total methionine oxidation in the reference standard during months 51–66, with investigation tracing the cause to the microcentrifuge tube bag. (**A**): Methionine oxidation in the reference standard of the Fc-fusion protein during a 7-year period, with observed methionine oxidation increasing over time during months 51–66. The oxidation level decreased to normal after a new bag of microcentrifuge tube was used after month 70. (**B**): Investigation performed in Lab 2 comparing devices (pipets and tubes) and reagents (denaturing and digestion buffer) used in the two labs. (**C**): Investigation performed in Lab 1 comparing devices (tubes) and reagents (iodoacetate) used in the two labs. Results from both labs revealed that the microcentrifuge tube used for collecting buffer-exchanged solution was the root cause. Each dot in (**A**–**C**) represents one experimental measurement. Vertical axes in (**B**,**C**) represent total methionine oxidation.

The Fc-fusion protein reference standard was analyzed in an analytical laboratory (“Lab 1”) over a 7-year period. During the trending over the 7-year period, apparent oxidation levels in the reference standard abruptly increased by up to ~5-fold during certain runs (Figure 2A). The reference standard sample was then analyzed in another laboratory (“Lab 2”) using the same method, and no increase was observed. The abrupt change persisted and increased across an extended period (months 51–66 in the trend record) and therefore could not be explained by random variability.

An investigation was initiated to identify the cause of the abrupt increase in oxidation levels observed in Lab 1. We suspected that the increased oxidation was the result of contaminants or impurities introduced by devices or reagents during sample preparation. After assessing various devices and reagents used by the two laboratories, it was concluded that the increased oxidation was caused by some unknown species from a particular bag of microcentrifuge tubes (Figure 2B,C) used for collecting buffer-exchanged solutions in Lab 1. Discontinuing the implicated tubes returned oxidation levels to normal (Figure 2A after 70 month time point). The exact molecular species responsible was not identified. However, the fact that artificial oxidation increased over time during months 51–66 (Figure 2A) suggested that it is related to the age of the microcentrifuge tube under the storage condition used in Lab 1. We suspect that the coating on the tubes may have been impacted by extended exposure to light, since the bag in question was kept under fluorescent ceiling lights for a prolonged period of time. This assessment, however, is speculative in nature and the true mechanism of the increased oxidation remains unknown.

**Figure 3:**
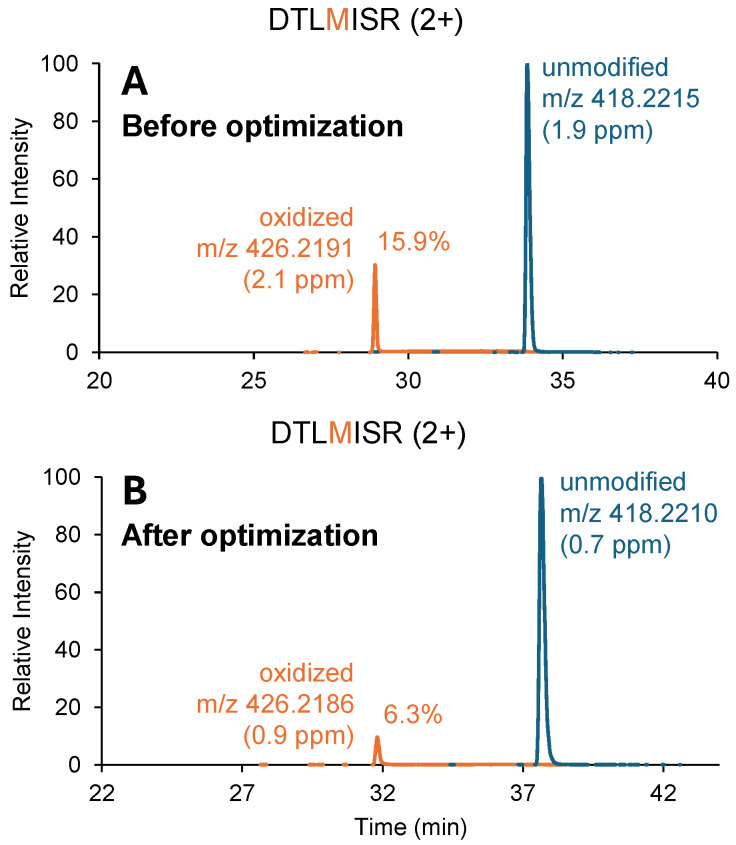
XICs for the conserved CH_2_ domain peptide, DTLMISR, from an IgG1 antibody–drug conjugate. (**A**): DTLMISR oxidation is observed to be artificially elevated up to 15.9% due to sample-preparation-associated oxidation. (**B**): Following method optimization, apparent DTLMISR oxidation is reduced to 6.3%. Theoretical monoisotopic *m*/*z* of the unmodified and oxidized peptides are 418.2207 and 426.2182, respectively.

During a different study examining elevated methionine oxidation in an antibody–drug conjugate (ADC), optimizing the sample preparation method led to a reduction in CH_2_ domain methionine oxidation from 15.9% to 6.3% (Figure 3). The optimized method included replacing Zeba™ spin desalting columns with NAP-5 gravity desalt columns and adding 20 mM L-methionine to the denaturation and quench buffers. Although the exact factor that caused the significant decrease in artificial oxidation is not clear, the method optimization focused on reducing the concentration of potential oxidizing species through better desalting and providing excess L-methionine in solution to act as a scavenger. To further validate the reduction in artificial oxidation by the optimized method, measurements were repeated 20 additional times using both methods. Methionine oxidation was observed at 12.1% ± 3.5% (mean ± standard deviation) prior to optimization, and at 7.2% ± 0.5% following optimization.

### 2.2. On-Column and Post-Column Oxidations

**Figure 4:**
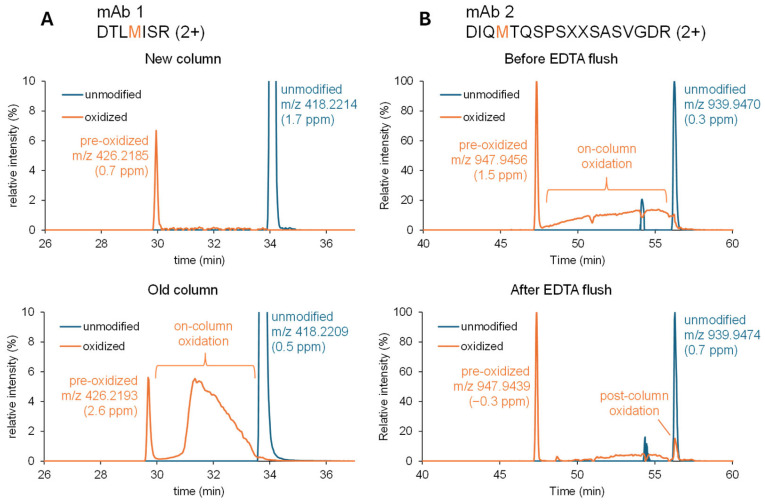
XICs illustrating (i) pre-oxidized peak (earliest), (ii) post-column oxidized signal coeluting with unmodified peptide, and (iii) broad on-column oxidation peak between them. (**A**): On-column oxidation increases with column age. XICs are normalized to the maximum intensity of the unmodified ion and zoomed at the bottom 10%. Theoretical monoisotopic *m*/*z* of the unmodified and oxidized peptides are 418.2207 and 426.2182, respectively. (**B**): On-column oxidation can be reduced by EDTA flushing. Each XIC is normalized to its maximum intensity. The additional peak at ~54 min for the unmodified trace is due to an interference of an unrelated peptide 2 Da smaller. Theoretical monoisotopic *m*/*z* of the unmodified and oxidized peptides are 939.9467 and 947.9442, respectively.

In one of the mAb molecules (mAb 1) monitored by MAM, the level of methionine oxidation in the reference standard exhibited high variability across long-term trending. Further investigation revealed that the variability was caused by on-column methionine oxidation that increased as the LC column aged (mAb 1, Figure 4A). When oxidation occurs during chromatographic migration, the random timing of oxidation yields a broad peak between the pre-oxidized and unmodified peptide peaks in the oxidized-peptide XIC [13]. This broad, noisy feature can mislead automated processing and compromise correct peak integration, causing variability in the result. It also biases quantitation by reducing the unmodified peak intensity, potentially inflating apparent oxidation even when the pre-oxidized peak is correctly integrated. Additionally, on-column oxidation may happen before the peptide starts to migrate, in which case the artificial oxidized species would elute at the same retention time as the pre-oxidized species, causing overestimation of oxidation.

To reduce on-column oxidation of another mAb molecule (mAb 2), the LC flow path was extensively flushed using a mobile phase containing EDTA. This significantly reduced on-column oxidation (Figure 4B), suggesting that metal ions, likely related to corrosion products in the LC flow path, contributed to the artifact. This finding aligns with prior reports that residual metals in LC systems can drive variability in methionine oxidation readouts during peptide mapping and that managing metal exposure is critical for robust oxidation measurement [14]. Mobile phases in these methods contain 0.1% trifluoroacetic acid (TFA). We hypothesize that corrosion results from prolonged exposure to low-pH mobile phases (0.1% TFA, ~pH 1.9). Consistent with this, we rarely observe significant on-column oxidation when 0.1% formic acid (~pH 2.7) instead of TFA is used in the mobile phases.

The oxidized species coeluting with the unmodified species shown in Figure 4B (mAb 2) is consistent with post-column oxidation, potentially induced by UV radiation in the detector flow cell, especially for diode array detectors where all wavelengths of light pass through the flow cell, and/or by the electrospray ionization process [15,16]. When the UV detector is bypassed in a 0.1% formic acid system, we rarely observe significant amounts of post-column oxidation.

## 3. Discussion

We broadly categorize the technical challenges in implementing MAM into four areas.

1. Sample preparation. Due to the complexity of sample preparation, including denaturation, reduction, alkylation, buffer exchange, and proteolytic digestion, the procedure often requires significant expertise and extensive training. These steps are ideally automated. Automation has improved reproducibility and throughput, and continued advances in auto-digestion protocols show promise for routine application [17,18,19,20,21].
2. Instrument–instrument variability. MS-based MAM often assumes that modified and unmodified peptides have the same response factor (ionization and detection), or at least consistent response between instruments. However, some chemical modifications may significantly change the charge, size or hydrophobicity of the peptides that may affect the ionization and ion-transfer efficiencies, making the response factor sensitive to ion source and other ion-transfer conditions. Ultimately, this issue is inevitable as new MS technologies render older instrument models obsolete. This problem, however, is considered solvable through run-time instrument calibration from a well characterized reference standard [22].
3. Data analysis. Quantifying a large number of attributes from LC-MS data often requires integration of hundreds of peaks, making manual verification impractical. As software continues to develop and become more sophisticated, this issue will ultimately be resolved. Progress has already been achieved in this area [4]. Another challenge is the complex task of new-peak detection [23], though advances have been made in statistical methods, [24] and the use of peak libraries [25,26,27] to reduce false positives while maintaining detection sensitivity.
4. Artificial modifications. Many critical quality attributes, such as deamidation, aspartic acid isomerization, and oxidation of methionine and tryptophan, can occur both during storage and as artifacts of sample preparation and analysis. These artificial modifications must be properly controlled for accurate quantitation of these attributes in the therapeutic protein. Well-controlled artifacts can be mathematically corrected using a standard as calibrant [22]. While modifications like deamidation and isomerization are primarily influenced by pH, temperature, and reaction time, and thus are controllable, oxidation is uniquely challenging. Factors such as trace metal ions, photosensitizers, peroxides, dissolved oxygen, and light exposure can induce oxidation, and these are difficult to control or even detect. Although data are not presented here, we anticipate that artificial oxidation of other residues such as Trp, Cys, His, and Tyr behaves similarly, as they are affected by similar environment factors. Our findings demonstrate that artificial oxidation can originate from unexpected sources, such as a specific bag of microcentrifuge tubes, and may not become apparent until a significant number of samples have been analyzed over time. Our results also demonstrate that optimizing sample preparation reduced observed methionine oxidation from 15.9% to 6.3%, underscoring the importance of method parameters to accurate quantitation. Furthermore, although the method was optimized to minimize artificial oxidation, it remains unclear to what extent the observed 6.3% is attributable to artificial oxidation. To determine the true methionine oxidation level, the method must be validated using a negative control (with minimal methionine oxidation) to demonstrate that it is capable of producing a negligible amount of artificial oxidation.

The challenge of artificial oxidation must be properly addressed, especially in a quality control setting, when timely release of clinical or commercial material is critical. If left unresolved, artificial oxidation can significantly delay the release of therapeutic products, as time-consuming investigations may be required to identify the root cause. Given its unpredictability and high impact [28], artificial oxidation, in our opinion, is the most critical barrier to the successful and reliable implementation of MAM in quality control environments.

We urge practitioners and researchers to focus their efforts on understanding and eliminating this artifact to ensure robust and efficient biotherapeutic analysis. One possible direction is to seek an appropriate reactive oxygen scavenger. Although L-methionine was added in the digestion buffer to scavenge reactive oxygen species, it was apparently not sufficient to eliminate oxidation artifact during sample preparation of the Fc-fusion protein. A stronger antioxidant is needed to eliminate artificial oxidation while preserving protein structure and proteolytic activity. In the microcentrifuge tube case, the causative chemical species was not identified. Future work should couple extractables/leachables chemistry with controlled oxidation assays to create predictive qualification tests for plastics and contact surfaces. Practitioners in the field are also encouraged to identify oxidation-promoting leachables from typical contact surfaces.

Before a protocol is developed with minimal artificial oxidation, proper controls must be implemented to ensure reliable measurement of methionine and tryptophan oxidation. A practical control framework for oxidation artifacts includes, but is not limited to:

1. Consumable qualification and change control. The microcentrifuge tube case shows that consumable-associated leachables can drive major oxidation bias. For oxidation-sensitive workflows, high-impact consumables (tubes, tips, filter devices, etc.) should be placed under change control and evaluated with an oxidation-sensitive probe (e.g., a reference digest or methionine-containing peptide) before introduction into regulated testing.
2. Reference standard system suitability. Parallel analysis of a frozen reference standard in every run provides a real-time readout of oxidation artifacts.
3. Metal management across the LC flow path and column lifecycle. Because on-column oxidation can increase with column age and be reduced by EDTA flushing, column lifecycle criteria should include oxidation-artifact metrics (appearance of broad peak) rather than only pressure and chromatographic resolution. Where feasible, adopting lower-metal flow paths or passivated components and scheduling conditioning/chelation steps can improve robustness.
4. Where method performance allows, consider replacing TFA with formic acid or lowering TFA concentration to reduce corrosion-driven metal exposure.
5. Bypass the UV detector when feasible to reduce post-column oxidation. If a UV detector is used, avoid a diode array detector to reduce overall light exposure.
6. Data-analysis safeguards for oxidation signatures. On-column oxidation produces a characteristic broad hump that can be used as a system suitability criterion [13].

## 4. Materials and Methods

### 4.1. Fc-Fusion Protein

An analytical method, based on tryptic digestion and high-resolution MS/MS, has been developed [5] for the multi-attribute analysis of the Fc-fusion protein (Amgen, Thousand Oaks, CA, USA). Before analysis, the reference standard sample was thawed at 2–8 °C and brought up to room temperature. Sample preparation includes sequential steps of denaturation, reduction, alkylation, and buffer exchange, followed by enzymatic digestion. Specifically, 200 µg of the Fc-fusion protein was first denatured by diluting (to a final volume of 500 µL) into a denaturing buffer containing 7.5 M guanidine HCl (MP Biomedicals, Santa Ana, CA, USA), 250 mM Tris (Teknova, Hollister, CA, USA), 2 mM EDTA (Thermo Fisher Scientific, Waltham, MA, USA) at pH 7.5; then its disulfides were reduced by adding 10 µL of 500 mM dithiothreitol (DTT) (Thermo Fisher Scientific Pierce) and incubating at room temperature for 30 min, followed by adding 20 µL of 500 mM sodium iodoacetate (MilliporeSigma, Burlington, MA, USA) and incubating at room temperature for 20 min. Alkylation was quenched by adding 20 µL of 50 mM DTT. Using a NAP-5 column (Cytiva, Wilmington, DE, USA), the reduced/alkylated sample was exchanged into a digestion buffer containing 100 mM Tris and 50 mM methionine (MilliporeSigma) at pH 7.5. The eluent from the NAP-5 column was collected into a microcentrifuge tube (Eppendorf Safe-Lock^®^ Tubes, 1.5 mL, Hauppauge, NY, USA), into which 6.6 µL of 1 mg/mL trypsin (Roche, South San Francisco, CA, USA) solution was added, followed by incubation at 37 °C for 30 min. The digestion was quenched by adding 100 µL of 250 mM acetate (J.T. Baker, Radnor, PA, USA) buffer (pH 4.7) containing 8 M guanidine HCl. Each analytical run includes parallel analysis of a reference standard for system suitability and trending purposes.

Each digest was analyzed on an LC-MS system containing an Agilent (Santa Clara, CA, USA) 1290 UHPLC system directly connected (without going through a UV/Vis detector) to a Thermo Fisher Scientific Q Exactive Plus high-resolution mass spectrometer. Tryptic peptides were separated on an Agilent Zorbax 2.1 × 150 mm C18, 1.8 µm particle size and RRHD column at 50 °C using an acetonitrile gradient at a flow rate of 0.25 mL/min. Mobile phases A and B are water and acetonitrile, respectively, each containing 0.1% formic acid. Peptide elution begins at 1% B for 5 min, increases linearly to 10% B over 3 min, then to 42.5% B over 67 min, followed by column washing and equilibration. Data analyses were performed on Thermo Fisher Scientific Chromeleon version 7.3.1 with integrated MS data processing to quantify attributes of interest using their standard workflow. Level of methionine oxidation is determined from the MS peak area of the oxidized peptide divided by the sum of peak areas of oxidized and unmodified peptides, assuming equal response factor between the oxidized peptide and un-oxidized peptide.

### 4.2. ADC

Prior to method optimization, the ADC protein (Amgen) was first diluted into 15 mM DTT. Then, 500 µg of the protein was denatured by diluting (to a final volume of 500 µL) into a denaturing buffer containing 7.5 M guanidine HCl, 250 mM Tris and 2 mM EDTA at pH 7.5, then its disulfide was reduced by adding 10 µL of 0.5 M DTT and incubating at 37 °C for 30 min, followed by adding 20 µL of 0.5 M sodium iodoacetate and incubating in the dark at room temperature for 30 min, and then followed by quenching the alkylation by adding 10 µL of 0.5 M DTT. The reduced/alkylated samples were exchanged into a 50 mM Tris digestion buffer (pH 7.5) using a Zeba™ column (Thermo Fisher Scientific). The eluent from the Zeba™ column was collected into a microcentrifuge tube and digested by trypsin at an ~1:10 enzyme:substrate ratio at 37 °C for 30 min. The digestion was quenched by addition of 5% TFA to a final concentration of 0.5%.

To reduce artificial oxidation, the method was optimized by diluting the protein directly into the denaturing buffer. An amount of 20 mM L-methionine was added into the denaturing buffer and quench solution to serve as an oxygen scavenger. In addition, a NAP-5 gravity size exclusion column instead of a Zeba™ column was used for buffer exchange, and the final quenched digest contained 0.1% TFA instead of 0.5%.

Each digest was analyzed on an LC-MS system containing an Agilent 1290 with a VWD, or Waters (Milford, MA, USA) Acquity I-Class with a TUV detector, connected to a Thermo Fisher Scientific Q Exactive Plus high-resolution mass spectrometer. Tryptic peptides were separated on a Waters BEH 2.1 × 150 mm C18, 1.7 µm particle size and column at 65 °C using an increasing acetonitrile gradient at a flow rate of 0.3 mL/min. Mobile phases A and B were water and acetonitrile, respectively, each containing 0.1% TFA. Data were analyzed using MassAnalyzer 6.07 [1], a custom program developed in-house (currently available in Biopharma Finder 5.4 from Thermo Fisher Scientific).

### 4.3. mAbs

All mAbs (Amgen) were analyzed using LC-MS workflows analogous to those employed for the Fc-fusion protein and ADC, with minor methodological differences between them. The target molecule was first denatured in a buffered guanidine solution (7.5–8 M, including Tris pH 7.5–8.0) and reduced by addition of DTT. Free thiol groups were then alkylated by addition of sodium iodoacetate and incubation protected from light. After the alkylation reaction was quenched by a second addition of DTT, the samples were exchanged into a Tris digestion buffer (pH 7.5–8.0) using NAP-5 size exclusion columns. Trypsin was added at a ~1:10 enzyme-to-protein mass ratio followed by incubation at 37 °C for 30 min prior to quenching by acidification.

Digests were separated by reversed phase chromatography on either a Waters Acquity I-Class or Agilent 1290 system using a Waters BEH 2.1 × 150 mm, 1.7 µm particle size C18 column heated to 50 °C with water (A) and acetonitrile (B) mobile phases, each acidified with 0.1% TFA by volume. The static flow rates utilized by each method varied from 0.2–0.25 mL/min. UV absorption was recorded at 214 nm by Waters TUV or Agilent VWD detectors and mass spectra recorded on an Orbitrap Q-Exactive Plus mass spectrometer. LC-MS/MS data were analyzed using MassAnalyzer 6.07 [1].

EDTA flushing (Figure 4B) was performed by purging the UHPLC system and column with 20 mM EDTA in a 10% acetonitrile water solution at 0.3 mL/min for 20 h. The system was then equilibrated with water and acetonitrile containing 0.1% TFA at 0.3 mL/min for 24 h before sample injection.

## 5. Conclusions

While multiple challenges exist in the implementation of MS-based multi-attribute methods for therapeutic protein characterization, artificial oxidation during sample preparation and analysis, in our opinion, stands out as the most significant and difficult to control. Addressing this issue is critical for enabling the robust, reliable, and timely release of biotherapeutic products. Continued research and innovation are essential to overcome this barrier and fully realize the potential of MAM in quality control and regulatory settings.

## Data Availability

The original contributions presented in this study are included in the article. Further inquiries can be directed to the corresponding author.

## Abbreviations

The following abbreviations are used in this manuscript:

LC	Liquid chromatography
MS	Mass spectrometry
MAM	Multi-attribute method
UHPLC	Ultra-high performance liquid chromatography
EDTA	Ethylenediaminetetraacetic acid
mAb	Monoclonal antibody
XIC	Extracted-ion chromatogram
UV	Ultraviolet
TFA	Trifluoroacetic acid
DTT	Dithiothreitol
ADC	Antibody–drug conjugate
VWD	Variable wavelength detector
TUV	Tunable ultraviolet
